# Effect of Ki-67 proliferation index on survival in large cell neuroendocrine carcinoma of the lung

**DOI:** 10.1590/1806-9282.20240398

**Published:** 2024-09-16

**Authors:** Mustafa Emre Duygulu, Esra Aşık, Mehmet Akif Tükenmez, Gizem Teoman, Atila Yıldırım, Evren Fidan

**Affiliations:** 1 Karadeniz Technical University, Farabi Hospital, Department of Medical Oncology – Trabzon, Turkey.; 2 Karadeniz Technical University, Farabi Hospital, Department of Medical Pathology – Trabzon, Turkey.; 3 Kahramanmaraş Necip Fazıl City Hospital, Department of Medical Oncology – Kahramanmaraş, Turkey.

**Keywords:** Neuroendocrine carcinoma, Lung cancer, Ki-67

## Abstract

**OBJECTIVE:**

Large cell neuroendocrine carcinoma of the lung is a rare type of lung cancer. There is a limited number of studies on clinical and histopathological characteristics that are effective in survival. The aim of this study was to investigate the relationship between histopathological and clinical characteristics, mainly Ki-67 proliferation index, and survival in patients diagnosed with large cell neuroendocrine carcinoma of the lung.

**METHODS:**

The data of 38 patients followed up with the diagnosis of large cell neuroendocrine carcinoma of the lung were evaluated. The mean Ki-67 value was determined to be 65.8% (±20.8). The patients’ clinical characteristics and survival times were compared according to the cut-off value determined for Ki-67 index.

**RESULTS:**

When median overall survival times were compared, it was seen that overall survival was numerically lower in patients aged 65 years and over, in tumors located on the right side, in cases who were in the metastatic stage at diagnosis, whose Ki-67 index was 65% and above, who did not receive chemotherapy, who did not undergo curative surgery, and in patients with chronic diseases (p>0.05). In the Kaplan-Meier analysis, the median overall survival was determined to be 22.2 months (95%CI 21.7–22.7) in the patients with Ki-67<65%, while it was found to be 20.3 months (95%CI 4.5–36.2) in the patients with Ki-67≥65% (p=0.351).

**CONCLUSION:**

Our study identified subgroups with decreased survival in large cell neuroendocrine carcinoma of lung patients. Studies including a larger number of patients are needed to identify the prognostic importance of these clinical and histopathological characteristics.

## INTRODUCTION

Large cell neuroendocrine carcinoma (LCNEC) of the lung is a rare type of lung cancer, and its incidence has been reported to be between 2.1 and 3.5%^1^. It is a high-grade histological subtype with a poor prognosis that displays rapid progression^2^. Histomorphologically, it has the common characteristics of non-small cell lung cancer (NSCLC) and small cell lung cancer (SCLC)^3^. Its aggressive tumor biology is similar to that of SCLC^4^. Due to its low prevalence, there is no randomized clinical study data for treatment options, and patient management is performed in a similar manner to SCLC cases. The number of studies revealing features associated with a poor prognosis in LCNEC patients is very limited.

Ki-67 is an antigen that is coded by the MKI67 gene, expressed in cell cycles outside the G0 phase, and reached its maximum level at the onset of the mitosis phase (M). Today, a high Ki-67 index is accepted as a proliferation marker that indicates a poor prognosis in many different cancer types^5-8^. There is no consensus yet on a Ki-67 value that has a diagnostic value or can be accepted as prognostic for LCNEC.

In this study, it was aimed to investigate the relationship between histopathological and clinical characteristics, mainly Ki-67 proliferation index, and survival in patients with the diagnosis of LCNEC of the lung.

## METHODS

### Patients

The clinical, demographic, and histopathological characteristics and overall survival (OS) of patients followed up at our center with the diagnosis of lung LCNEC between January 2010 and November 2023 were retrospectively evaluated. The data were obtained from the electronic system of the hospital. The inclusion criteria were determined as being 18 years old and above, the primary tumor origin being the lung, the Ki-67 index being numerically evaluated in the pathology report, and access to patient data. The exclusion criteria were determined as being diagnosed with an additional malignancy, the origin of the primary tumor being in an organ other than the lung, the Ki-67 index not being numerically evaluated in the pathology report, and the inability to access patient data.

### Evaluation of the Ki-67 index

Formalin-fixed, paraffin-embedded tissue blocks were used for immunohistochemistry staining. The anti-Ki-67 (Ventana, 30-9) rabbit monoclonal primary antibody was used on the Ventana BenchMark ultra automated slide stainer platform. Tumor cells that showed Ki-67 nuclear protein expression of any intensity were counted as positive cells. The Ki-67 proliferative index was calculated by dividing the number of Ki-67 positive tumor cells by the total number of counted tumor cells.

The Ki-67 index was evaluated by the pathologist using the eyeballing method. In this approach, the pathologist identified the region exhibiting hot spot staining. Cells displaying nuclear staining with Ki-67 in the hot spot were counted within an area containing a total of 500 tumor cells. The ratio of positively stained tumor cells to the total count of 500 tumor cells was reported as the Ki-67 index.

### Statistical method

For statistical analyses, NCSS (Number Cruncher Statistical System) 2007 (Kaysville, Utah, USA) software was employed. In the evaluation of the study data, descriptive statistical methods (mean, standard deviation, median, frequency, ratio, minimum, maximum) were used. The Mann-Whitney U test and the Kruskal-Wallis Test were used in group comparisons. In the comparison of qualitative data, the Pearson chi-square test was used. OS was calculated using the Kaplan-Meier method. The statistical significance value was determined as p<0.05.

Karadeniz Technical University Faculty of Medicine Ethics Committee approval, dated 29.02.2024 and protocol number 2023/271, was obtained before the study. The principles of the Declaration of Helsinki were followed at all stages of the study.

## RESULTS

In total, 38 patients were evaluated within the scope of the study. The number of male patients was 37 (97%), while the number of female patients was 1 (3%). The median age was determined to be 64 (min 48–max 79). The number of patients under the age of 65 years was 20 (52.6%), and the number of patients at the age of 65 years and above was found to be 18 (47.4%). Considering the study of Ishibashi et al., the mean value was used for the Ki-67 cut-off value^9^. The Ki-67 mean value was determined to be 65.8% (±20.8). The number of patients with a Ki-67 value lower than 65% was found to be 16 (42.1%), and the number of those with a Ki-67 value of 65% and above was 22 (57.9%). The ECOG (Eastern Cooperative Oncology Group) performance of the majority of the patients (94%) was between 0 and 2. The percentages of the patients according to their characteristics were as follows: 73% had a tumor located in the right lung, 68.4% were in T1–T2 stage at the diagnosis, 71.1% were in stage N0–N1 at the diagnosis, 76.3% were in M0 stage at the diagnosis, 84.2% had pure large cell neuroendocrine histology, 84.2% had received adjuvant/palliative chemotherapy, and 68.4% had undergone curative surgery (Table 1).

**Table 1:** Evaluation of clinical and histopathological findings according to Ki-67 groups.

Variable		Total n (%)	Ki-67 65%< n (%)	Ki-67 65%≥ n (%)	p-value
ECOG	ECOG 0–2	36 (94.7)	15 (93.8)	21 (95.5)	0.816*
	ECOG 3–4	2 (5.3)	1 (6.3)	1 (4.5)	
Primary tumor side	Left	10 (26.3)	5 (31.3)	5 (22.7)	0.556*
	Right	28 (73.7)	11 (68.8)	17 (77.3)	
T stage (TNM-8)	1	14 (36.8)	6 (37.5)	8 (36.4)	0.331*
	2	12 (31.6)	6 (37.5)	6 (27.3)	
	3	8 (21.1)	4 (25.0)	4 (18.2)	
	4	4 (10.5)	0 (0)	4 (18.2)	
N stage (TNM-8)	0	18 (47.4)	10 (62.5)	8 (36.4)	0.400*
	1	9 (23.7)	3 (18.8)	6 (27.3)	
	2	10 (26.3)	3 (18.8)	7 (31.8)	
	3	1 (2.6)	0 (0)	1 (4.5)	
M stage (TNM-8)	0	29 (76.3)	12 (75.0)	17 (77.3)	0.871*
	1	9 (23.7)	4 (25.0)	5 (22.7)	
Accompanying histology^1^	Absent	32 (84.2)	12 (75.0)	20 (90.9)	0.184*
	Present	6 (15.8)	4 (25.0)	2 (9.1)	
Adjuvant/palliative chemotherapy	Absent	6 (15.8)	4 (25.0)	2 (9.1)	0.184*
	Present	32 (84.2)	12 (75.0)	20 (90.9)	
Adjuvant/palliative radiotherapy	Absent	20 (52.6)	11 (68.8)	9 (40.9)	0.090*
	Present	18 (47.4)	5 (31.3)	13 (59.1)	
Curative surgery	Absent	12 (31.6)	5 (31.3)	7 (31.8)	0.970*
	Present	26 (68.4)	11 (68.8)	15 (68.2)	
Diabetes mellitus	Absent	28 (73.7)	12 (75.0)	16 (72.7)	0.875*
	Present	10 (26.3)	4 (25.0)	6 (27.3)	
Hypertension	Absent	19 (50.0)	8 (50.0)	11 (50.0)	0.995*
	Present	19 (50.0)	8 (50.0)	11 (50.0)	
Chronic lung disease	Absent	29 (76.3)	13 (81.3)	16 (72.7)	0.542*
	Present	9 (23.7)	3 (18.8)	6 (27.3)	
Chronic heart disease	Absent	26 (68.4)	10 (62.5)	16 (72.7)	0.503*
	Present	12 (31.6)	6 (37.5)	6 (27.3)	
Death	Absent	17 (44.7)	7 (43.8)	10 (45.5)	0.917*
	Present	21 (55.3)	9 (56.3)	12 (54.5)	

* Pearson chi-square test

1 Adenocarcinoma (n=3). Squamous cell carcinoma (n=2). Small cell carcinoma (n=1). ECOG: Eastern Cooperative Oncology Group.

**Figure 1:**
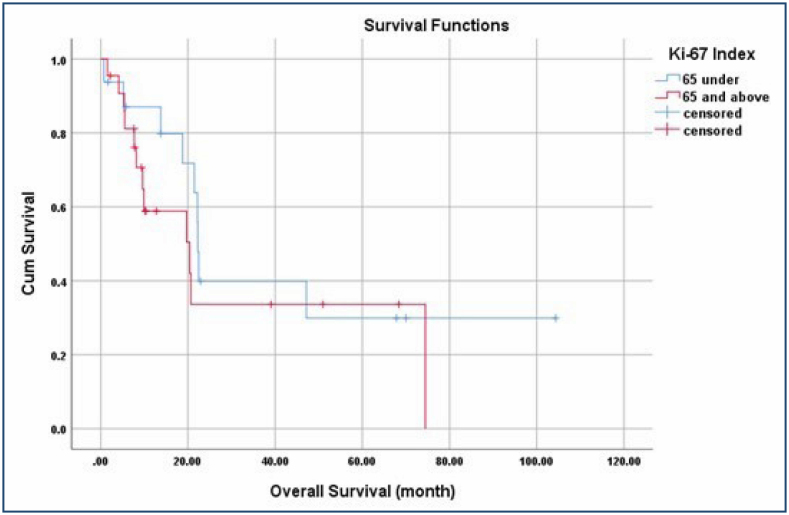
Overall survival curves of patients according to Ki-67 value.

When the patients’ clinical features were evaluated according to the Ki-67 mean value, no statistically significant difference was identified between the groups (Table 1). The median OS was determined to be lower in patients at the age of 65 years and above, in tumors located in the right lung, in patients with T4 tumor, with N2-3 disease, in patients in the metastatic stage at the diagnosis, with a Ki-67 index of 65% and above, in patients who did not receive chemotherapy, and with a comorbid disease (p>0.05) (Table 2). In Kaplan-Meier analysis, the median OS was calculated as 21.4 months (95%CI 18.6–24.2) in the whole population, 22.2 months (95%CI 21.7–22.7) in patients with Ki-67<65%, and 20.3 months (95%CI 4.5-36.2) with Ki-67≥65% (p=0.351) (Figure 1).

**Table 2:** Evaluation of overall survival times according to clinical and histopathological findings.

Variable		Percentile 25–75 (Median OS)	p-value
Age	65<	5.52–30.98 (20.04)	b0.492
	65≥	7.68–21.46 (9.75)	
ECOG performance status	ECOG 0–2	6.72–22.68 (13.27)	b0.728
	ECOG 3–4	7.68–18.79 (13.24)	
Primary tumor side	Left	9.32–67.82 (17.52)	b0.230
	Right	5.66–21.82 (12.06)	
T stage (TNM-8)	1	5.21–20.39 (10.29)	c0.445
	2	8.54–43.13 (17.61)	
	3	7.9–36.71 (20.52)	
	4	6.54–15.15 (8.65)	
N stage (TNM-8)	0	5.82–50.96 (13.27)	c0.932
	1	9.89–20.39 (18.79)	
	2	5.39–39.07 (10.29)	
	3	9.61–9.61 (9.61)	
M stage (TNM-8)	0	7.68–39.07 (13.75)	b0.184
	1	5.39–19.68 (9.61)	
Accompanying histology*	Absent	6.72–30.66 (10.29)	b0.379
	Present	19.68–22.46 (21.82)	
Ki-67 index	<65%	9.79–35.04 (21.82)	b0.145
	≥65%	7.61–20.39 (9.75)	
Adjuvant/palliative chemotherapy	Absent	5.21–22.46 (5.61)	b0.262
	Present	7.97–22.57 (13.75)	
Adjuvant/palliative radiotherapy	Absent	9.61–22.68 (19.24)	b0.128
	Present	5.39–21.46 (8.9)	
Curative surgery	Absent	6.5–18.11 (8.9)	b0.182
	Present	7.75–22.89 (19.24)	
Diabetes mellitus	Absent	7.93–30.98 (13.75)	b0.273
	Present	5.39–20.68 (8.82)	
Hypertension	Absent	8.18–47.18 (20.39)	b0.231
	Present	5.5–22.25 (9.89)	
Chronic lung disease	Absent	7.61–22.25 (13.75)	b0.986
	Present	8.18–22.89 (9.61)	
Chronic heart disease	Absent	7.75–47.18 (13.75)	b0.340
	Present	5.61–20.97 (10.13)	

b Mann-Whitney U test.

c Kruskall-Wallis test.

* Adenocarcinoma (n=3). Squamous cell carcinoma (n=2). Small cell carcinoma (n=1). ECOG: Eastern Cooperative Oncology Group.

## DISCUSSION

In the study, the clinical and histopathological data of patients diagnosed with LCNEC of lung were evaluated. Median OS was compared in the whole population according to their clinical and histopathological characteristics. OS was found to be lower, though not statistically significant but considerable in clinical practice, in patients with a Ki-67 proliferation index of 65% and above, whose primary tumor was located in the right lung, who were in the metastatic stage at the diagnosis, who did not receive adjuvant/palliative chemotherapy, who did not undergo curative surgery, and who had a chronic disease (diabetes mellitus, hypertension, chronic lung disease, chronic heart disease).

The World Health Organization (WHO) has categorized lung neuroendocrine carcinomas into four categories: typical carcinoids, atypical carcinoids, SCLC, and LCNEC cancer. It has been stated that a 30% cut-off value in the Ki-67 index can be an indicator of neuroendocrine carcinomas. In addition, the diagnostic contribution of the Ki-67 index to the distinction between neuroendocrine tumors and carcinoma in biopsy preparations with crush artifact has been reported^10,11^. While a 20% cut-off value is used in the distinction of neuroendocrine carcinomas in the neuroendocrine tumors of the gastrointestinal system, there is no such diagnostic cut-off value for lung neuroendocrine tumors^12^.

Large cell neuroendocrine carcinoma is a subtype that proliferates fast and has a poor prognosis, resulting in decreased survival. It is prevalent in the male gender^13^. As studies in this regard are limited, an approach similar to SCLC treatment is adopted in the treatment of LCNEC. The standard treatment consists of surgical and adjuvant platin–etoposide combination therapy in the early stages and palliative platin–etoposide therapy in the metastatic stage. In studies conducted, 5-year OS rates for all stages have been reported between 15 and 52%^14^. In a study conducted on 2,368 LCNEC patients over Surveillance, Epidemiology, and End Results (SEER) data, advanced age, male gender, increased tumor diameter, presence of lymph node metastasis, presence of distant organ metastasis, absence of surgical intervention, and not receiving radiotherapy or chemotherapy were evaluated as poor prognostic factors that affect survival^15^. In our study, OS times were found to be lower in the subgroups of patients similar to the mentioned study, the differences are that survival times were lower in patients with a high Ki-67 index, tumors located on the right side, and patients diagnosed with chronic diseases.

Due to limited information, the importance of the Ki-67 value in choosing treatment options in patients diagnosed with LCNEC, determining patient groups at risk, and its place in treatment selection or prognosis determination are not clearly known yet. There are few studies investigating the effect of the Ki-67 index on survival in lung LCNEC. In a multicenter study conducted by Milione et al. including 111 lung LCNEC and combined LCNEC cases where all patients received curative or palliative surgical treatment, it was determined that a Ki-67 index of 55% and above is a poor prognostic marker. In the same study, advanced age, central tumor location, and Napsin-A staining negativity were also evaluated as poor prognostic markers^16^. Similarly, in the present study, OS times were found to be numerically lower in the patient group with a high Ki-67 index. In our study, approximately 30% of the patients had not undergone curative surgery, and the pure LCNEC patient ratio was higher.

The most significant limitations of the present study were the limited number of patients resulting from the single-center experience and the rarity of LCNEC in the lung.

## CONCLUSION

As a result of the study, clinical and histopathological characteristics associated with decreased survival times in patients diagnosed with LCNEC were identified and shared as a single-center experience. We believe that with studies involving a larger number of patients, the prognostic importance of these factors in terms of survival can be investigated more comprehensively.
